# Supplementary material for: Population-level impacts of antibiotic usage on the human gut microbiome
Source: Nat Commun. 2023 Mar 2;14:1191. doi: 10.1038/s41467-023-36633-7 (PMC9981903; doi:10.1038/s41467-023-36633-7)
Supplement: Supplementary file 1 — Supplementary Information [file 41467_2023_36633_MOESM1_ESM.pdf]

Supporting information

Supplementary Tables

			Health status[1]			Antibiotic exposure[2]			Healthy & not on antibiotic
Country (ISO)	Continent	Total	Health	Disease	NA or special cases	No	Yes	NA	
Madagascar (MDG)	Africa	112	112			112			112
Tanzania (TZA)		55			55[T]			55	
Bangladesh (BGD)	Asia	30	4	26		13	17		4
China (CHN)		1,296	340	620	336	1008	5	283	340
Israel (ISR)		956	956			956			956
Kazakhstan (KAZ)		168	168			168			168
Mongolia (MNG)		110			110			110	
Austria (AUT)	Europe	154	16	138		154			16
Denmark (DNK)		403	401	2		403			401
France (FRA)		157	62	95		157			62
Germany (DEU)		268	182	82	4	109		159	103
Italy (ITA)		42	42			33	1	8	33
Netherlands (NLD)		520	470	50		520			470
Spain (ESP)		356	209	147		139		217	139
Sweden (SWE)		299	204	91	4	200		99	109
United Kingdom (GBR)		250			250			250	
Canada (CAN)	North America	312	75	237		36	250	26	36
United States of America (USA)		465	191	170	46 58[N]	147		318	147
Fiji (FJI)	Oceania	133	133					133	
Peru (PER)	South America	18			18[T]			18	
Total		6,104	3,565	1,658	881	4,155	273	1,676	3,096

Table S1: **Metagenome datasets used in this study for the population-level analyses of the gut antibiotic resistome.** The table gives the number of adult stool metagenome samples from each country that were used in the analyses of country-level correlation to antibiotic consumption rate and inter-personal variation of gut resistome. [1] Health status was determined from the description of the original study subject recruitment criteria, and on the sample's categorization into disease-control grouping whenever the original study was presented in a diseased-versus-healthy comparison scheme. The 'disease' label here encompasses several different unhealthy states including CDI, cholera, colorectal adenoma, CRC, IBD, liver cirrhosis, T2D, fatty liver, hypertension, rheumatoid arthritis, and STEC infection. See Figure S11 for full disease acronym definitions. [2] We labelled the sample with 'No' for 'Antibiotic Exposure' 'if the original metadata description explicitly indicated that the given sample's donor was not taking any form of antibiotic at the time of sample collection, or if the original study's recruitment criteria included the exclusion of subjects that had been taking antibiotics within a defined period preceding the enrollment. [T] Populations with traditional lifestyle, [N] Noncontemporary samples.

Clustering threshold		Number of clusters					
		Total	Including microbiome ORFs	Including only microbiome ORFs	Including RefSeq ORFs	Including only RefSeq ORFs	Including both microbiome and RefSeq
**ARG_cluster90:**							
90% identity 80% coverage		9,467	2,215	399	9,068	7,252	1,816
	(% of total catalogue)		23.4%	4.21%	95.8%	76.6%	19.2%
	(% of microbiome)			18.0%			82.0%
	(% of RefSeq)					80.0%	20.0%
**ARG_cluster95:**							
95% identity 80% coverage		17,180	3,758	1,097	16,083	13,422	2,661
	(% of total catalogue)		21.9%	6.39%	93.6%	78.1%	15.5%
	(% of microbiome)			29.2%			70.8%
	(% of RefSeq)					83.5%	16.5%
**ARG_cluster99:**							
99% identity 90% coverage		55,472	15,907	9,683	45,789	39,565	6,224
	(% of total catalogue)		28.7%	17.5%	82.5%	71.3%	11.2%
	(% of microbiome)			60.9%			39.1%
	(% of RefSeq)					86.4%	13.6%
**ARG_cluster100:**							
100% identity 90% coverage		247,406	65,260	50,744	196,662	182,146	14,516
	(% of total catalogue)		26.4%	20.5%	79.5%	73.6%	5.87%
	(% of microbiome)			77.8%			22.2%
	(% of RefSeq)					92.6%	7.38%

Table S2: **Clustering of ARG catalogue.** All ARG ORFs collected from the metagenome assemblies of the human microbiome dataset and RefSeq genome assemblies were pooled and clustered by average nucleotide identity. Clustering was performed with the cascaded clustering workflow using a greedy set cover algorithm, according to the default settings of the cluster command in the program mmseqs2 at four different nucleotide identity and coverage cut-offs (see Methods).

Country	Number of usable samples (read-based)	ARG abundance (cpg)			Country level richness of ARG_cluster95 in subsampled dataset								
					Raw reads (Gbp) in picked samples			Number of picked samples			Number of ARG_cluster95		
		Median	Q1	Q3	Median	Q1	Q3	Median	Q1	Q3	Median	Q1	Q3
CHN	340 (209)	3.86	2.94	5.65	100.1	98.9	100.8	26	25	27	222	205	240
ITA	33 (33)	2.76	1.77	3.94									
MDG	112 (112)	3.15	1.84	6.96									
USA	147 (115)	2.80	2.10	3.67	100.9	98.0	102.8	10	10	10	55	45	81
FRA	62 (62)	5.44	3.28	6.77	99.7	98.7	101.0	19	19	21	177	168	196
ESP	139 (139)	5.56	4.09	8.18	100.3	98.7	101.6	16	15	16	157	144	182
KAZ	168 (168)	3.60	2.44	5.23	100.0	98.6	101.5	17	17	18	145	133	162
DEU	103 (103)	1.95	1.47	2.65	100.0	99.4	100.6	40	38	42	57	53	72
ISR	956 (937)	3.56	1.82	11.30	100.0	99.1	100.9	34	32	36	141	125	167
SWE	109 (109)	1.52	1.17	2.39	100.1	96.4	105.5	8	7	10	114	100	129
DNK	401 (230)	2.83	1.70	4.54	99.8	98.2	101.4	18	17	19	126	116	146
CAN	36 (35)	1.70	1.34	2.62	101.1	97.5	103.5	8	7	8	86	57	99
AUT	16 (16)	1.55	1.11	2.02									
NLD	470 (468)	1.08	0.81	1.60	100.1	99.2	101.0	33	32	34	71	57	96

Table S3: **Country level comparison of the abundance and diversity of ARGs in healthy individuals.** Stool metagenomes sampled from healthy adults who were not taking antibiotics were used to calculate the country-based population level statistics on the abundance and diversity of ARGs. Only countries with at least 10 samples that met the criteria were analyzed. We used read-based profiling to derive median total abundance of ARGs (copies per genome cpg) for each country. Diversity of ARGs was calculated by randomly picking subsamples from each country until the total raw reads in the picked samples amount to 100 ± 10 Gbp and counting the number of ARG_cluster95 discovered in the subsamples. The subsampling was repeated 99 times. Countries that had a total of less than 100 Gbp of raw reads from the eligible individuals according to the above criteria were excluded from this analysis. The resulting richness estimates were scaled to the number of clusters per 100 Gbp to adjust for slight variations in the actual size of subsampled datasets. The median, the first quartile (Q1), and the third quartile (Q3) values are presented for each parameter.

AMU data source	Value type	Resistome parameter<br>Scope of ARGs and/or gene diversity unit	Countries tested<br>Number	 Outlier	Shapiro-Wilks<br>p	Kendall<br>tau	 p	 FDR	Pearson<br>r	 p	 FDR
CDDEP	Abundance from reads	Total	12	CHN	6.7E-2	0.63	**5.7E-3 ***	**1.9E-2 ***	0.89	**2.3E-4 ***	**8.3E-3 ***
	Abundance based on assembled catalog	Total	12	CHN	6.8E-1	0.49	**4.1E-2 ***	7.4E-2	0.80	**2.9E-3 ***	**1.7E-2 ***
		Plasmid ORFs			2.5E-1	0.64	**5.7E-3 ***	**1.9E-2 ***	0.84	**1.1E-3 ***	**1.3E-2 ***
		Non-plasmid ORFs			1.0E+0	0.53	**2.6E-2 ***	5.2E-2	0.77	**5.9E-3 ***	**1.9E-2 ***
		Multi-species clusters			6.0E-1	0.60	**9.9E-3 ***	**2.5E-2 ***	0.83	**1.6E-3 ***	**1.4E-2 ***
		Single-species clusters			**3.5E-2 ***	0.26	2.9E-1	3.1E-1	0.45	1.7E-1	2.0E-1
		LCA-unassigned clusters			**2.9E-4 ***	0.30	2.2E-1	2.5E-1	0.09	8.0E-1	8.0E-1
WHO	Abundance from reads	Total	10	None	7.0E-2	0.60	**1.7E-3 ***	**9.3E-3 ***	0.65	**4.0E-2 ***	7.6E-2
	Abundance based on assembled catalog	Total	10	None	4.8E-1	0.73	**2.2E-3 ***	**9.9E-3 ***	0.82	**3.9E-3 ***	**1.8E-2 ***
		Plasmid ORFs			1.8E-1	0.69	**4.7E-3 ***	**1.9E-2 ***	0.84	**2.3E-3 ***	**1.7E-2 ***
		Non-plasmid ORFs			4.6E-1	0.64	**9.1E-3 ***	**2.5E-2 ***	0.71	**2.2E-2 ***	**5.0E-2 ***
		Multi-species clusters			2.1E-1	0.64	**9.1E-3 ***	**2.5E-2 ***	0.75	**1.2E-2 ***	**3.1E-2 ***
		Single-species clusters			8.2E-2	0.45	7.8E-2	1.3E-1	0.40	2.5E-1	2.6E-1
		LCA-unassigned clusters			**1.5E-4 ***	0.31	2.3E-1	2.5E-1	0.10	7.8E-1	8.0E-1
CDDEP	Richness	ARG_cluster90, total	10	CHN	6.5E-1	0.42	1.2E-1	1.7E-1	0.57	1.1E-1	1.4E-1
		ARG_cluster95, total			6.6E-1	0.39	1.8E-1	2.1E-1	0.57	1.1E-1	1.4E-1
		ARG_cluster99, total			4.8E-1	0.39	1.8E-1	2.1E-1	0.63	6.7E-2	1.0E-1
		ARG_cluster100, total			4.3E-1	0.39	1.8E-1	2.1E-1	0.62	7.7E-2	1.1E-1
WHO	Richness	ARG_cluster90, total	8	None	7.4E-1	0.86	**1.7E-3 ***	**9.3E-3 ***	0.85	**7.3E-3 ***	**2.0E-2 ***
		ARG_cluster95, total			8.4E-1	0.86	**1.7E-3 ***	**9.3E-3 ***	0.86	**6.3E-3 ***	**1.9E-2 ***
		ARG_cluster99, total			3.7E-1	0.86	**1.7E-3 ***	**9.3E-3 ***	0.87	**5.5E-3 ***	**1.9E-2 ***
		ARG_cluster100, total			6.7E-1	0.71	**1.4E-2 ***	**3.2E-2 ***	0.80	**1.8E-2 ***	**4.3E-2 ***
CDDEP	Richness	ARG_cluster99, multi-species	10	CHN	4.0E-1	0.44	1.2E-1	1.7E-1	0.63	6.8E-2	1.0E-1
		ARG_cluster99, single-species			4.0E-1	0.39	1.8E-1	2.1E-1	0.59	9.1E-2	1.3E-1
		ARG_cluster99, LCA-unassigned			5.5E-1	0.20	4.6E-1	4.6E-1	0.47	2.0E-1	2.3E-1
WHO	Richness	ARG_cluster99, multi-species	8	None	6.6E-1	0.71	**1.4E-2 ***	**3.2E-2 ***	0.69	6.1E-2	1.0E-1
		ARG_cluster99, single-species			8.0E-1	0.86	**1.7E-3 ***	**9.3E-3 ***	0.93	**9.5E-4 ***	**1.3E-2 ***
		ARG_cluster99, LCA-unassigned			6.2E-1	0.47	1.1E-1	1.7E-1	0.46	2.5E-1	2.6E-1
CDDEP	Richness	ARG_cluster95, from plasmid	10	CHN	**4.5E-2 ***	0.25	3.5E-1	3.6E-1	0.70	**3.7E-2 ***	7.4E-2
		ARG_cluster99, from plasmid			**6.7E-2 ***	0.40	1.4E-1	1.9E-1	0.65	5.6E-2	1.0E-1
		ARG_cluster95, from non-plasmid			4.3E-1	0.42	1.2E-1	1.7E-1	0.52	1.5E-1	1.8E-1
		ARG_cluster99, from non-plasmid			5.4E-1	0.44	1.2E-1	1.7E-1	0.63	6.8E-2	1.0E-1
WHO	Richness	ARG_cluster95, from plasmid	8	None	1.5E-1	0.69	**1.8E-2 ***	**3.8E-2 ***	0.74	**3.5E-2 ***	7.4E-2
		ARG_cluster99, from plasmid			4.6E-1	0.62	**3.4E-2 ***	6.4E-2	0.61	1.1E-1	1.4E-1
		ARG_cluster95, from non-plasmid			2.9E-1	0.91	**1.8E-3 ***	**9.3E-3 ***	0.87	**5.3E-3 ***	**1.9E-2 ***
		ARG_cluster99, from non-plasmid			6.7E-1	0.93	**4.0E-4 ***	**9.3E-3 ***	0.88	**3.7E-3 ***	**1.8E-2 ***

Table S4: **Country level correlation between total antibiotic consumption rate and gut resistome prevalence and richness.** Country median gut resistome profiles were derived from the subjects that were identifiable as healthy adults that were currently unexposed to antibiotics at the time of sampling. Median relative abundance of ARGs was calculated only for the countries that had at least 10 samples that met the criteria. ARG richness was calculated only for the countries where at least 100 Gbp total sequencing reads were collected from the subjects that met the criteria. The resulting richness estimates were scaled to the number of clusters per 100 Gbp to adjust for slight variations in the actual size of subsampled datasets. Total antibiotic consumption rates in the units of - defined daily does - DDD per 1,000 capita per year was used. P-values and Benjamini-Hochberg-adjusted FDR values below 0.05 are highlighted (*).

	CDDEP consumption data						WHO consumption data					
	Spearman			Kendall			Spearman			Kendall		
Antibiotic	rho	p	FDR	tau	p	FDR	rho	p	FDR	tau	p	FDR
**Median abundance of ARG summed by antibiotic class (cpg) vs. consumption rate of the antibiotic class**												
Aminoglycoside	0.18	0.57	0.99	0.09	0.74	0.91	-0.03	0.93	0.93	-0.07	0.79	0.79
Beta-lactam	0.50	0.099	0.59	0.42	0.063	0.38	**0.73**	**0.021***	0.13	**0.56**	**0.029***	0.17
Tetracycline	-0.11	0.73	0.99	-0.09	0.74	0.91	-0.26	0.47	0.70	-0.20	0.48	0.73
Quinolone	-0.20	0.53	0.99	-0.18	0.47	0.91	0.14	0.70	0.84	0.11	0.70	0.79
MLSB	0.01	0.99	0.99	-0.06	0.84	0.91	0.30	0.41	0.70	0.20	0.48	0.73
Amphenicol	-0.03	0.94	0.99	-0.03	0.91	0.91	0.51	0.13	0.40	0.40	0.15	0.46
**Richness of ARG_cluster99 in 100 Gbp subsample of metagenomes from the country vs. consumption rate of the antibiotic class**												
Aminoglycoside	0.20	0.58	0.91	0.11	0.73	0.73	0.07	0.87	0.87	0.04	0.90	0.9
Beta-lactam	0.15	0.68	0.91	0.16	0.60	0.73	0.50	0.22	0.6	0.29	0.40	0.8
Tetracycline	-0.55	0.10	0.63	-0.47	0.073	0.44	-0.19	0.66	0.87	-0.14	0.72	0.9
Quinolone	-0.04	0.91	0.91	-0.09	0.72	0.73	0.46	0.26	0.6	0.25	0.38	0.8
MLSB	0.05	0.89	0.91	0.11	0.73	0.73	0.43	0.30	0.6	0.43	0.18	0.8
Amphenicol	0.18	0.64	0.91	0.13	0.66	0.73	-0.08	0.85	0.87	-0.07	0.83	0.9

Table S5: **Country level correlation between antibiotic consumption rate in each antibiotic class versus median abundance and rarefied richness of the corresponding ARGs in the gut metagenomes.** Six broad antibiotic classes that were covered by both WHO and CDDEP statistics were used in the analyses. Abundance of ARG families in each sample were summarized at the level of antibiotic classes based on the annotation given in the CARD database. Only stool samples from subjects that were identifiable as healthy adults that were currently unexposed to antibiotics at the time of sampling were included in the analysis. Median abundance of ARGs in the countries that have at least 10 samples were tested for correlation with the defined daily dose (DDD per 1,000 per year) statistics from WHO or CDDEP. Richness of ARG_cluster99s were calculated for each country for the ARG_cluster99s affiliated to each target antibiotic class. From each country, random selections of samples was performed to reach 100 $\pm$ 5 Gbp of total sequence reads with 100 iterations, and the number of ARG_cluster99s in the resulting subsamples were scaled to the number of clusters per 100 Gbp to adjust for slight variations in the actual size of subsampled datasets. Median number of clusters per 100 Gbp derived from 100 iterations represented the richness at country level. P-values or Benjamini-Hochberg-adjusted FDR values below 0.05 were highlighted ($*$).

	ARG_cluster99						ORF			
	Multi species		Single species		Unassigned		Binned		Unbinned	
	N	%	N	%	N	%	N	%	N	%
**Antibiotic class**										
**Sulfonamide**	10	47.6	2	9.5	9	42.9	157	6.8	2142	93.2
**Diaminopyrimidine**	34	19.5	58	33.3	82	47.1	595	12.0	4358	88.0
**Aminoglycoside**	137	18.6	281	38.1	319	43.3	3117	25.8	8948	74.2
**Beta-lactam**	87	15.6	168	30.2	302	54.2	3731	26.7	10221	73.3
**Glycopeptide**	85	14.2	117	19.6	396	66.2	1486	17.9	6834	82.1
**Amphenicol**	47	13.7	55	16.1	240	70.2	361	8.1	4117	91.9
**Tetracycline**	188	11.4	253	15.3	1212	73.3	2792	10.5	23832	89.5
**MLSB**	207	10.2	444	21.9	1379	67.9	3056	14.5	17958	85.5
**Fosfomycin**	23	5.3	234	53.9	177	40.8	1190	56.7	909	43.3
**Peptide**	72	4.7	876	57.6	574	37.7	5634	62.2	3423	37.8
**Multi-drug**	293	4.4	3543	52.7	2891	43.0	33980	62.1	20726	37.9
**Fluoroquinolone**	31	3.1	336	33.4	640	63.6	1990	65.6	1044	34.4
**Other classes**	20	20.0	35	35.0	45	45.0	172	12.4	1217	87.6
**Resistance mechanism**										
**Target replacement**	46	23.4	60	30.5	91	46.2	755	10.4	6501	89.6
**Inactivation**	305	19.0	408	25.5	889	55.5	6181	17.7	28786	82.3
**Target protection**	159	15.4	239	23.2	632	61.4	3032	14.5	17916	85.5
**Target alteration**	208	7.5	970	35.0	1594	57.5	6947	26.5	19314	73.5
**Efflux pump**	423	5.1	3756	44.9	4195	50.1	30046	55.1	24495	44.9
**Regulator**	92	4.8	969	50.3	864	44.9	11299	56.4	8717	43.6

Table S6: **Proportion of multi-species ARG_cluster99s charted by the antibiotic class the genes confer resistance to or the resistance mechanism**. Only the ARG_cluster99s that contain one or more ORFs from adult gut metagenomes (n = 6,104) were counted in this table. Classification of the ARG clusters into the categories (antibiotic class and resistance mechanism) was made based on the annotation of the representative ORF of the cluster. The annotations of the ARG ORFs were derived from the CARD database (see Methods).

(A) Antibiotic resistance class							
		Mean of $\log_{10}$ cpg			Mann-Whitney	
Resistotype	Resistance class	Major	Minor-FAMP	Diff.	P	FDR
Background	Glycopeptide	-1.8	-2.0	0.22	6.6E-16	1.6E-15
	Tetracycline	0.066	-0.024	0.090	1.3E-6	2.3E-6
FAMP	MDR	-3.9	-0.29	3.62	0	0
	Peptide	-4.2	-0.67	3.48	0	0
	Fosfomycin	-4.6	-2.1	2.46	0	0
	Fluoroquinolone	-4.4	-1.4	3.0	0	0
	Aminoglycoside	-2.1	-0.97	1.14	2.0E-215	7.5E-215
	Sulfonamide	-3.8	-2.8	1.08	2.1E-140	6.8E-140
	MLSB	-0.74	-0.52	0.22	2.0E-23	5.5E-23
	Rifamycin	-4.6	-4.5	0.050	7.8E-13	1.6E-12
	Beta.lactam	-0.68	-0.49	0.19	2.2E-12	4.1E-12
	Amphenicol	-2.5	-2.4	0.13	9.0E-4	1.4E-3
	Triclosan	-4.6	-4.6	0.013	1.2E-3	1.8E-3

(B) Species										
					Mean of $\log_{10}$ composition			Mann-Whitney	
Resistotype	SGB ID	Label	Preval. in the healthy	Pathogen?	Major	Minor-FAMP	Diff.	p	FDR
Background	4809	s__Blautia_SGB4809	0.56	No	-2.9	-3.3	0.92	8.0E-41	7.8E-39
	5117	s__Coprococcus_eutactus	0.92	No	-2.4	-2.6	0.88	4.8E-60	1.1E-57
	4828	s__Blautia_SGB4828	0.69	No	-2.8	-3.1	0.88	1.4E-53	2.3E-51
	4931	s__Lachnospiraceae_SGB4931	0.79	No	-2.5	-2.7	0.88	1.2E-36	8.9E-35
	4198	s__Eubacterium_siraeum	0.70	No	-2.5	-2.8	0.86	5.1E-30	2.8E-28
	4829	s__Blautia_SGB4829	0.48	No	-3.1	-3.4	0.83	1.2E-33	8.4E-32
	2318	s__Alistipes_SGB2318	0.87	No	-2.0	-2.1	0.83	1.2E-24	4.6E-23
	4648	s__Roseburia_SGB4648	0.69	No	-3.1	-3.3	0.82	4.2E-49	5.6E-47
	2290	s__Alistipes_obesi	0.86	No	-2.5	-2.6	0.80	2.1E-22	6.9E-21
	1965	s__Barnesiella_intestinihominis	0.77	No	-2.6	-2.8	0.80	1.5E-30	8.6E-29
FAMP	10067	s__Escherichia_coli	0.24	No	-4.6	-2.3	4.4	0	0
	9992	s__Proteus_mirabilis	0.24	Yes	-4.6	-2.5	4.2	0	0
	10066	s__Escherichia_fergusonii	0.21	Yes	-5.0	-2.7	3.9	0	0
	10131	s__Enterobacter_cloacae	0.24	Yes	-5.2	-3.0	3.9	0	0
	10068	s__Escherichia_coli	0.61	Yes	-3.5	-2.0	3.8	0	0
	10119	s__Klebsiella_michiganensis	0.20	No	-5.1	-2.9	3.6	0	0
	15630	s__Mycobacterium_avium	0.19	Yes	-5.4	-3.1	3.4	0	0
	7852	s__Staphylococcus_aureus	0.21	Yes	-4.6	-2.8	3.3	0	0
	10115	s__Klebsiella_pneumoniae	0.19	Yes	-4.5	-2.7	3.2	0	0
	22989	s__Escherichia_SGB22989	0.12	No	-5.9	-3.2	2.3	4.5E-305	2.1E-302

Table S7: **Resistance classes and species enriched in the resistotypes (A) Normalized abundance of ARGs (cpg) was summed by the antibiotic class and compared between the two resistotypes.** Stool metagenomes assigned to one of the two resistotypes (n = 5,372) were used. A pseudo-abundance value of 2.5e-5 cpg, the smallest non-zero value in the matrix of ARG relative abundances, was added before log transformation. Resistance classes with Benjamini-Hochberg-adjusted FDR value < 0.05 following two-sided Mann-Whitney tests were included in the table. (B) Compositional abundance of species-level genome bins (SGBs) was compared between the two resistotypes. A pseudo-abundance value of 1.02E-9 was added before the log transformation. Stool metagenomes assigned to resistotypes were used except for the samples that were not profiled at species level (< 1,000 ORFs recovered for single-copy core COGs). SGBs were labelled as pathogen or not based on the 463 pathogen names identified from the reference (see Methods). Detection frequency of each SGB among the healthy adults who were not taking antibiotics (n = 3,096) was given as 'preval. in the healthy'.

Enterotype	SGB	Fold	FDR	Species	Genus	Family	Phylum
1 “Lachnospiraceae”	4953	5.74	1.6E-195	*Roseburia* sp. CAG_182	*Roseburia*	*Lachnospiraceae*	Firmicutes
	4289	5.69	1.5E-110	*Eubacterium* sp. OM08_24	*Eubacterium*	*Eubacteriaceae*	Firmicutes
	4670	5.68	3.3E-219	*Coprococcus catus*	*Coprococcus*	*Lachnospiraceae*	Firmicutes
	4328	5.68	3.4E-66	Firmicutes bacterium CAG_341	Firmicutes unc.	Firmicutes unc.	Firmicutes
	714	5.65	1.9E-112	*Methanobrevibacter smithii*	*Methanobrevibacter*	*Methanobacteriaceae*	Euryarchaeota
	4809	5.65	2.4E-197	*Blautia* SGB4809	*Blautia*	*Lachnospiraceae*	Firmicutes
	14625	5.56	1.1E-148	*Collinsella* SGB14625	*Collinsella*	*Coriobacteriaceae*	Actinobacteria
	6778	5.56	1.7E-115	*Catenibacterium mitsuokai*	*Catenibacterium*	*Erysipelotrichaceae*	Firmicutes
	14624	5.52	4.2E-140	*Collinsella* SGB14624	*Collinsella*	*Coriobacteriaceae*	Actinobacteria
	14574	5.52	4.9E-125	*Collinsella* sp. AF28_5AC	*Collinsella*	*Coriobacteriaceae*	Actinobacteria
2 “Prevotella”	1613	7.52	0.0E+00	*Prevotella* sp. CAG_386	*Prevotella*	*Prevotellaceae*	Bacteroidetes
	1624	7.41	0.0E+00	*Prevotella* SGB1624	*Prevotella*	*Prevotellaceae*	Bacteroidetes
	1657	7.35	0.0E+00	*Prevotella* sp. CAG_1092	*Prevotella*	*Prevotellaceae*	Bacteroidetes
	1644	7.33	0.0E+00	*Prevotella* sp. TF12_30	*Prevotella*	*Prevotellaceae*	Bacteroidetes
	1701	6.90	0.0E+00	GGB1267 SGB1701	GGB1267	*Prevotellaceae*	Bacteroidetes
	1680	6.77	0.0E+00	*Prevotella* sp. CAG_520	*Prevotella*	*Prevotellaceae*	Bacteroidetes
	1699	6.68	1.1E-199	*Prevotella* sp.	*Prevotella*	*Prevotellaceae*	Bacteroidetes
	1684	6.64	4.5E-299	*Prevotella* sp. CAG_924	*Prevotella*	*Prevotellaceae*	Bacteroidetes
	1560	6.46	2.1E-232	*Prevotella intermedia*	*Prevotella*	*Prevotellaceae*	Bacteroidetes
	1677	6.29	3.3E-253	*Prevotella* sp. 885	*Prevotella*	*Prevotellaceae*	Bacteroidetes
3 “Bacteroides”	1890	6.16	5.1E-105	GGB1385 SGB1890	GGB1385	*Bacteroidaceae*	Bacteroidetes
	5792	6.08	2.3E-87	*Phascolarctobacterium faecium*	*Phascolarctobacterium*	*Acidaminococcaceae*	Firmicutes
	15132	5.95	4.8E-225	*Flavonifractor plautii*	*Flavonifractor*	Clostridiales unc.	Firmicutes
	1930	5.72	2.3E-79	*Parabacteroides* sp. CAG_409	*Parabacteroides*	*Tannerellaceae*	Bacteroidetes
	1831	5.71	2.0E-66	*Bacteroides* sp. AM10_21B	*Bacteroides*	*Bacteroidaceae*	Bacteroidetes
	7050	5.69	5.0E-47	*Lactobacillus amylovorus*	*Lactobacillus*	*Lactobacillaceae*	Firmicutes
	1870	5.57	1.1E-105	*Bacteroides* sp. D2	*Bacteroides*	*Bacteroidaceae*	Bacteroidetes
	15143	5.38	1.1E-209	*Flavonifractor* sp.	*Flavonifractor*	Clostridiales unc.	Firmicutes
	1785	5.32	5.2E-131	*Odoribacter* sp. AF21_41	*Odoribacter*	*Odoribacteraceae*	Bacteroidetes
	1940	5.28	9.6E-109	*Parabacteroides* sp. AF18_52	*Parabacteroides*	*Tannerellaceae*	Bacteroidetes

Table S8: **Species enriched in the enterotypes.** For each of three enterotypes, we show the top ten species that are strongly enriched in the given enterotype against the other two enterotypes. Species composition matrix based on single-copy core gene ORFs was used as input data. Wilcoxon rank sum test was performed on each species for each given enterotype. Cases with Benjamini-Hochberg-adjusted p-value < 0.05 were selected and ordered by the fold difference between the mean compositional abundance in the target and nontarget enterotypes, to give the top 10 species.

(A) Pathogenicity vs. Residency in healthy adult gut		
	**Pathogen**	**Non-pathogen**
**Gut resident**	27	717
**Infrequent colonizer**	210	3,732
**(B) Pathogenicity vs. Association with resistotype**		
	**Pathogen**	**Non-pathogen**
**Background type-associated**	0	27
**FAMP type-associated**	14	20
**Resistotype-nonspecific**	223	4,402
**(C) Residency in healthy adult gut vs. Association with resistotype**		
	**Gut resident**	**Infrequent colonizer**
**Background type-associated**	27	0
**FAMP type-associated**	30	4
**Resistotype-nonspecific**	687	3,938

Table S9: **Number of species-level genome bins counted according to the categorizations based on pathogenicity, gut residency, and resistotype association.** We defined the species as human pathogen if it was described in the Manual of Clinical Microbiology (American Society of Microbiology, Eleventh edition) as a confirmed agent in human infections of any type. We defined the species as gut resident if the frequency of its detection among the stool metagenomes from healthy adults not taking antibiotics (n = 3,096) was 10% or greater. We defined the species as associated with one of the two resistotypes when the means of the compositional abundance of the SGB was different by five-fold or greater and the Benjamini-Hochberg-adjusted p value was below 0.05 based on Mann-Whitney test.

Supplementary Figures

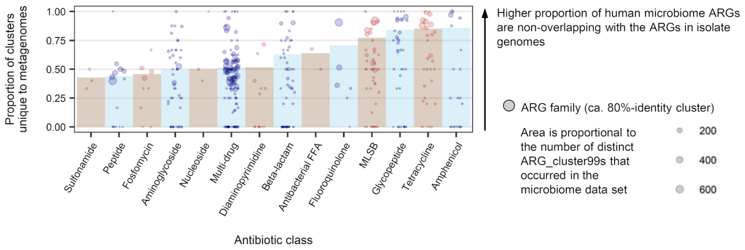

Figure S1: **Proportion of clusters non-overlapping with the isolate genome database for each ARG family categorised by antibiotic class.** Clusters were generated with a threshold of 99 percent nucleotide identity (ARG_cluster99) from the ARG sequences pooled from human microbiome metagenomic ORFs (sample n = 8,972, regardless of the body site; ORF n = 216,849) and NCBI RefSeq prokaryotic genomic ORFs (genome n = 152,407; ORF n = 2,349,728). Each cluster was assessed for inclusion of microbiome and RefSeq ORFs. Clusters without RefSeq ORFs were considered unique to the metagenome dataset. Each ARG family was then assessed for the proportion of its member ARG_cluster99s that are unique to metagenomes. ARG families were mapped to the antibiotic classes to which they confer resistance according to the CARD database. Semi-transparent bar plots in the background display the overall proportion of metagenome-unique clusters for each antibiotic class. Each of the bubble-shaped points visualizes an individual ARG family. We altered the colour scheme between antibiotic classes to aid visual distinction between classes.

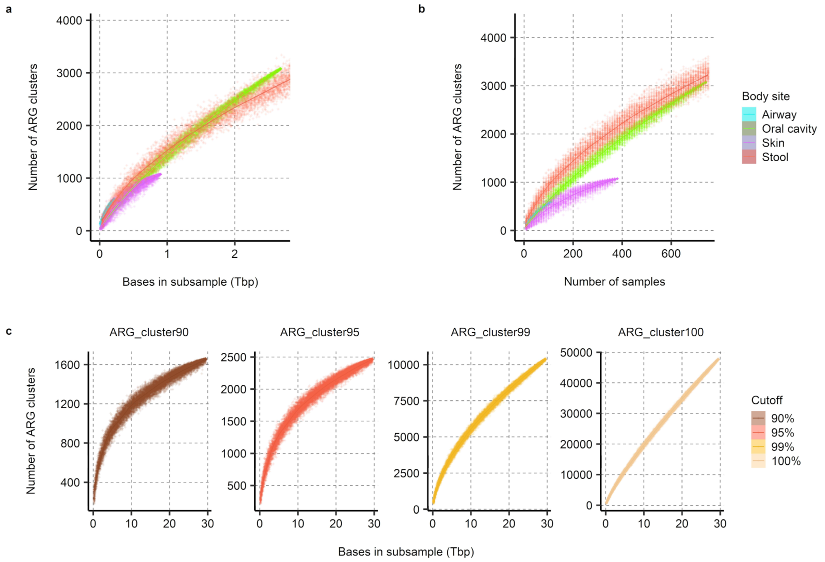

Figure S2: **The number of ARG clusters discovered with varying depth of subsampling compared across body sites.** (a) ARG_cluster99: subsample sizes expressed as the total amount of read bases in the subsamples. (b) ARG_cluster99: subsample sizes expressed as the number of subsamples. (c) Number of clusters as a functional of sequencing depth at different cut-offs for the gut only. Smoothed lines were generated by the gam function of mgcv as implemented in the R library ggplot2. In (a) and (b) the x-axis was trimmed at the maximum sample size of oral cavity, as displaying the full sample size of stool would dwarf the other body sites.

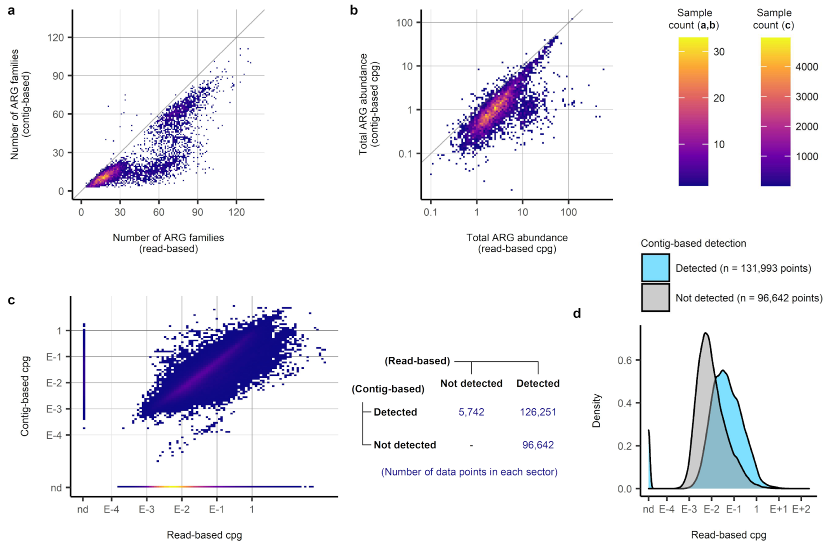

Figure S3: **Comparison between assembly-based and read-based profiling of ARGs.** (a-b) Two dimensional heatmaps estimating sample distributions. Data points were generated from 5,341 adult stool samples analyzed by both methods. (a) Sample distribution of the number of ARG families detected per sample with read-based (x-axis) and assembly-based (y-axis) methods. (b) Sample distribution of the total ARG abundance estimated per sample with read-based (x-axis) and assembly-based (y-axis) methods. (c) Individual cpg estimations for an ARG family in a sample using read-based (x-axis) and assembly-based (y-axis) methods were compared. Data points originated from 228,635 occurrences of any ARG family throughout the adult stool metagenomes within either read- or assembly-based method. (d) Density plots comparing read-based cpg value distributions of the ARG family occurrences according to whether the occurrence was detected or not detected using the assembly-based method.

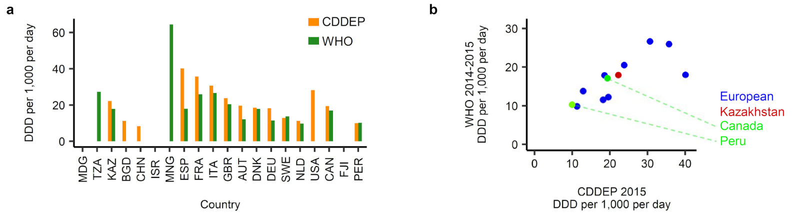

Figure S4: **Country level antibiotic consumption rate data from CDDEP and WHO.** (a) The x-axis lists the 20 countries for which we obtained gut resistome profiles from at least 10 samples. Countries are ordered based on continent. If antibiotic consumption rate data was available from either source for a country then it is shown as a bar on the y-axis as defined daily dose (DDD) per 1,000 (capita) per day summed across all antibiotics. (b) Scatter plot of the 12 countries that were covered by both CDDEP and WHO data. Pearson's correlation $r = 0.77$, $p = 0.0036$.

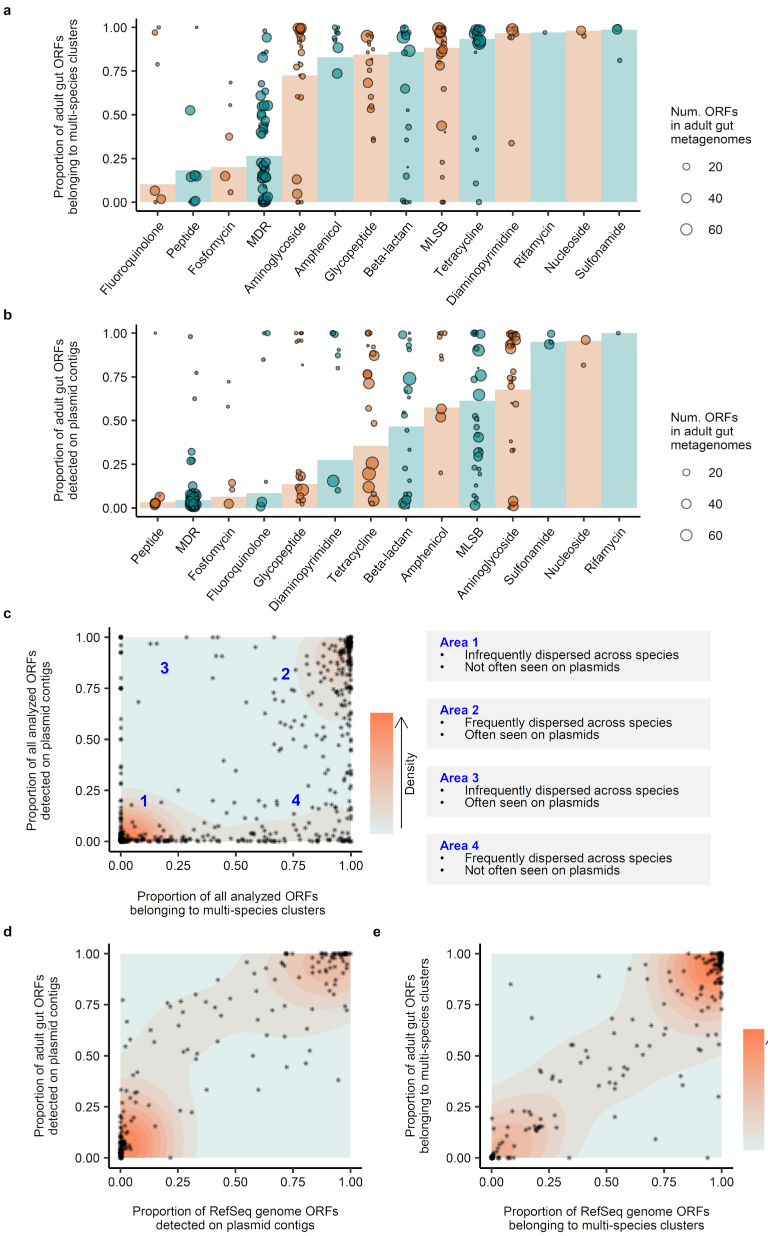

Figure S5: **Comparison of plasmid-borne and multi-species cluster distributions and assignments.** (a) Scatter-plot of ARG families giving the proportion of ORFs in multi-species clusters (x-axis) and the proportion of ORFs on plasmid contigs (y-axis). (b) Proportions of metagenomic ORFs detected on plasmid contigs plotted by the antibiotic class the genes confer resistance to. (c) Proportions of metagenomic ORFs attributable to multi-species clusters in each ARG family plotted by the antibiotic class the genes confer resistance to. Colors alternated simply to aid visual recognition. Vertical bars on the background show the antibiotic class-wide proportion. (d) Scatter plot of ARG families giving the proportion of ORFs on plasmid contigs estimated for the RefSeq genomic ORFs (x-axis) and the gut metagenomic ORFs (y-axis). (e) Scatter plot of ARG families giving the proportion of ORFs in multi-species clusters estimated for the RefSeq genomic ORFs (x-axis) and the gut metagenomic ORFs (y-axis).

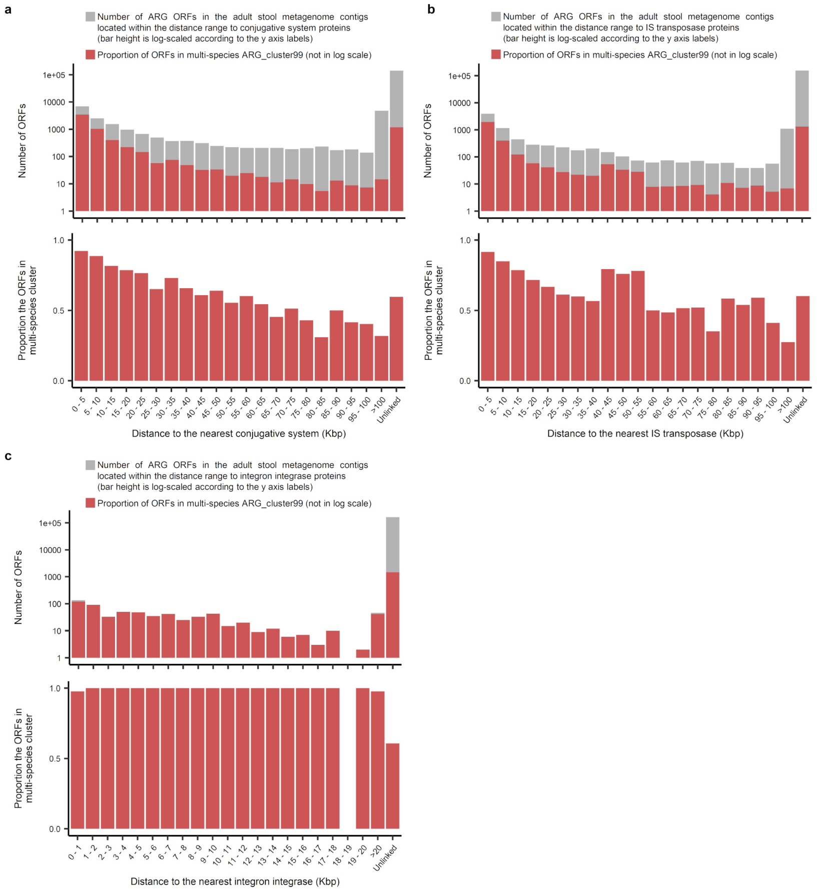

Figure S6: **Proportion of multi-species cluster ORFs according to their proximity to the marker genes of mobile genetic elements.** For each of three types of mobile genetic elements (MGEs), we grouped the ARG ORFs based on the distance to the nearest MGE marker genes found on the same contig. The number and the proportion of ORFs that belong to multi-species clusters (99%-identity) were plotted for each interval. 'Unlinked' is when there is not a target MGE marker gene on the same contig. (a) Distance to the conjugative system marker genes as defined in ConjDB. (b) Distance to the insertion sequence element transposases as defined in the ISFinder. (c) Distance to the integron integrases using the target references (AAQ16665.1, AAT72891.1, AAO32355.1, and 99031763) and outgroup references (P0A8P6.1 and P0A8P8.1). For conjugative systems and IS transposases (a, b) we used distance bins ranging up to 100 Kbp, since the sizes of known conjugative mobile genetic elements typically span 20 - 100 Kbp [1] and composite transposons up to 80 Kbp [2]. For integron integrases (c) we used shorter intervals for the distance bins, up to 20 Kbp, as the majority of integrons in bacterial genomes have a length from 5'-CS to 3'-CS lower than 10 Kbp [3].

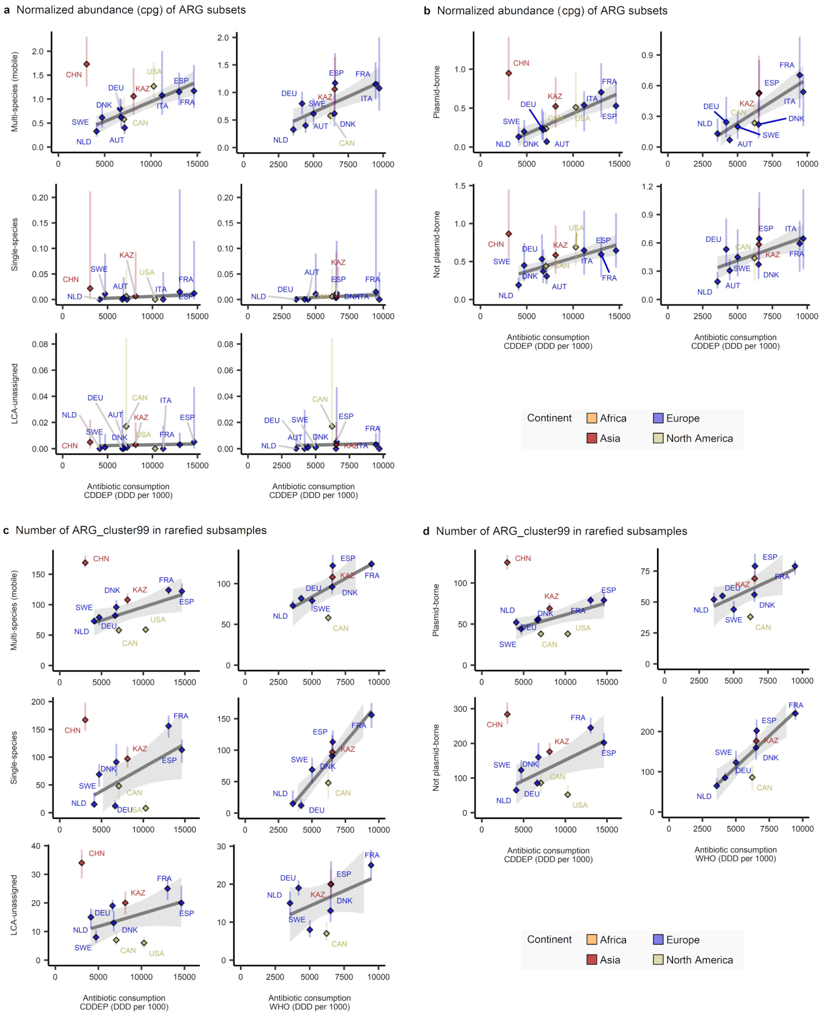

Figure S7: **Country level correlation between antibiotic consumption rates versus median abundance and richness of ARGs across gene mobility categories.** In (a,b) we display correlations between the median abundance (cpg) of ARGs in each gene mobility category and the antibiotic consumption rate (DDD per 1,000 per year) across the countries. (a) Using gene mobility categorization based on LCA assignments on ARG_cluster99. (b) Using gene mobility categorization based on PlasmidNet. In (c,d) we display correlations between the number of ARG_cluster99s recovered from rarefied subsamples (100 ± 10 Gbp per country) and the antibiotic consumption rates (DDD per 1000 per year) across the countries. (c) Using gene mobility categorization based on LCA assignments on ARG_cluster99. (d) Using gene mobility categorization based on PlasmidNet. Each panel is split with separate plots for CDDEP and WHO consumption rate data. Vertical lines indicate the range from the first quartile to the third quartile in per- sample ARG abundance (cpg) or the number of ARG_cluster99. Pairwise correlation tests were performed, excluding China from the CCDEP correlations (see Table S4 for the correlation test statistics). The number of metagenome samples used to derive the median and the range bars shown for each country in A and B: AUT (n = 16), CAN (n = 36), CHN (n = 340), DEU (n = 103), DNK (n = 401), ESP (n = 139), FRA (n = 62), ITA (n = 33), KAZ (n = 168), NLD (n = 470), SWE (n = 109), USA (n = 147). The number of rarefactions performed to derive each box plot and range bar shown in C and D: n = 99.

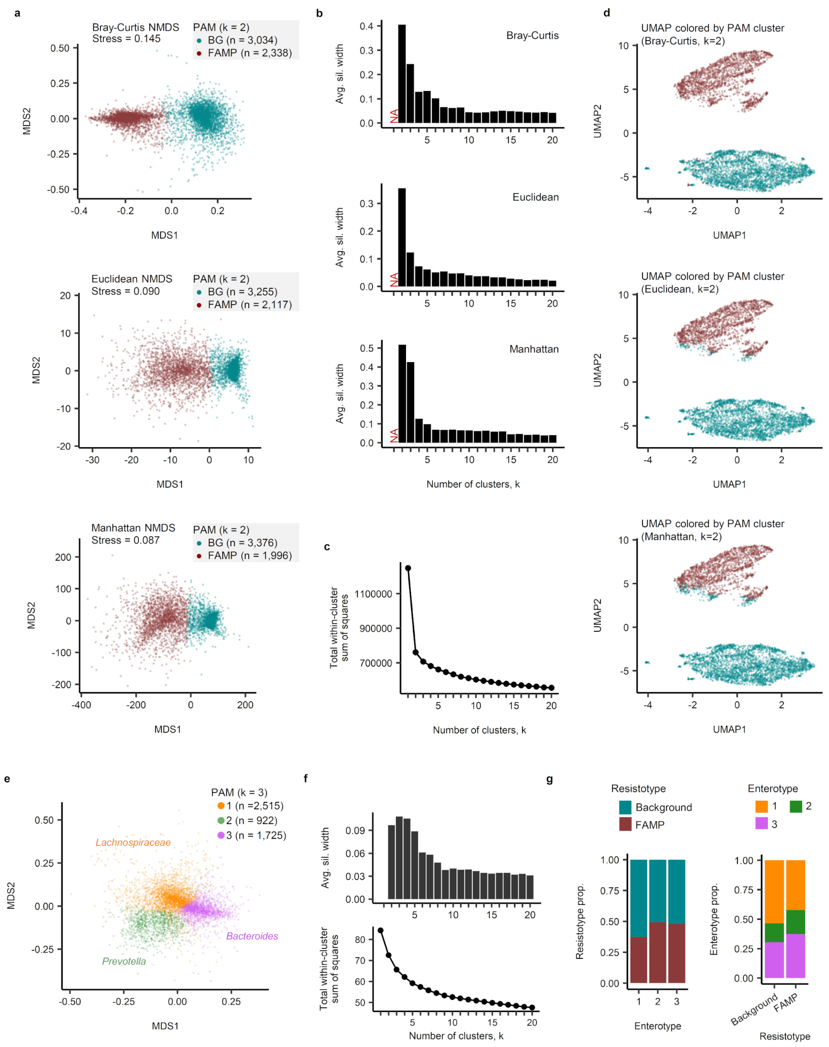

Figure S8: **Clustering of adult gut resistome profiles and species compositions.** The log-transformed ARG family profiles (cpg) of 5,372 non-outlier adult gut metagenomes in which three or more ARG families were detected, used as input for Figure 3A, was also used here throughout (a) – (e). (a) NMDS projection of resistome profiles based on three different dissimilarity measures, with a sample color scheme corresponding to the partitioning-around-medoid (PAM) clustering at k = 2. (b) Average silhouette width assessed after running PAM clustering on three different multivariate dissimilarity measures, i.e., Bray-Curtis, Euclidean, and Manhattan, with various pre-defined number of clusters, k. (c) Elbow plot, showing the total within-cluster sum of squares resulting from k-means clustering using various k from 1 to 20. (d) UMAP projection of resistome profiles with a sample color scheme corresponding to the PAM clusterings at k = 2 shown in the panel (a). (e) NMDS projection of species compositional profiles based on Bray-Curtis dissimilarity metric. Samples were colored according to PAM clustering with k value of 3. (f) Optimal k value for PAM clustering screened with an average silhouette width plot and an elbow plot. (g) Cross counts between the two resistotypes and the three enterotypes. Enterotype numbering corresponds to what are shown in the panel (f): 1, Blautia sp.; 2, Bacteroides sp.; 3, Prevotella copri.

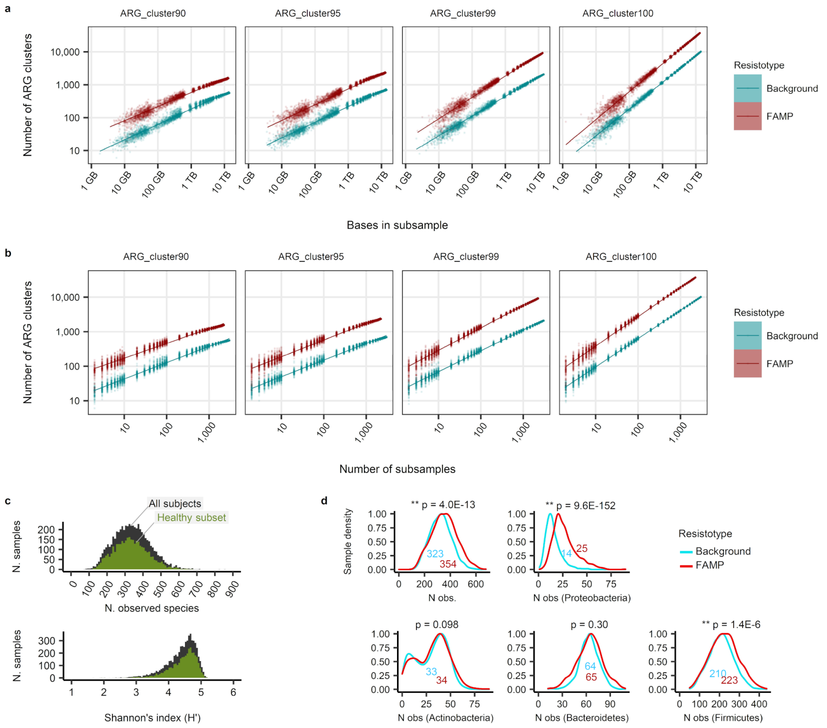

Figure S9: **ARG diversity and species diversity of the two resistotypes.** (a-b) Rarefaction curves of ARG clusters in the background and FAMP resistotype population. (a) Sample size on the x-axis expresses the total amount of bases in the raw sequence reads. (b) Sample size on the x-axis expresses the number of samples analyzed. All axes are in log scale. Fitting lines were drawn using a linear model using ggplot2 R package. (c-d) Species alpha-diversity of the stool metagenomes. (c) The number of observed species and Shannon's index (H') in the adult stool metagenomes that were subjected to resistotype analysis. The estimates are based on the species composition profiling based on the assembled ORFs that represent universal single-copy core COGs (see Methods). (d) Comparison of the distribution of the number of observed species in the background and the FAMP resistotypes. We repeated comparison by restricting the species counts to each of the four dominant phyla in the human gut microbiome. Comparisons were restricted to the samples from healthy subjects who were not taking antibiotics. The numbers displayed inside the plot area are the median value of each resistotype. P values displayed over the plots were determined by two-sided Wilcoxon rank sum tests.

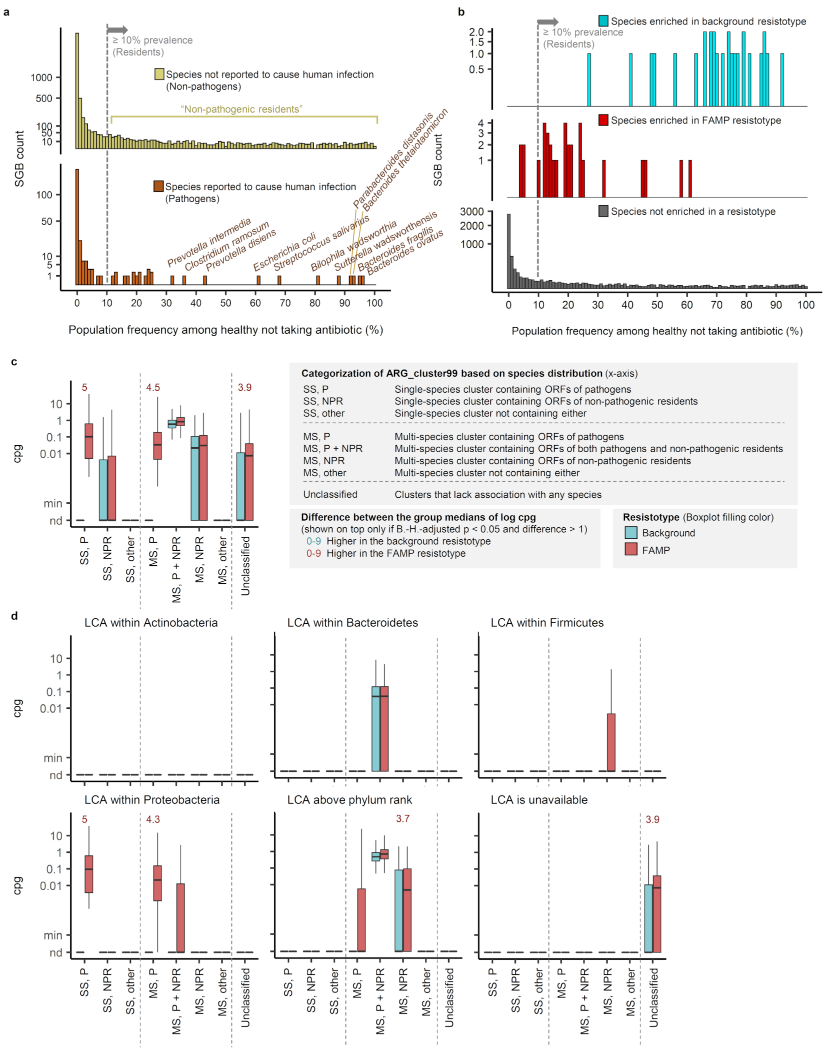

Figure S10: **Stratification of the differences between background and FAMP resistotypes based on the species association detected for ARG clusters.** (a) Species-level genome bins (SGBs) in the stool metagenomes are categorized into pathogens and non-pathogens and into the residents and infrequent colonizers. (b) Enrichment in a resistotype was defined as a median abundance at least five-fold greater in one resistotype than the other and a Benjamini-Hochberg adjusted $p < 0.05$ using two-sided Mann-Whitney test. In (a) and (b) the population level frequency of each SGB was calculated as the proportion of positive samples among the stool metagenomes from healthy adult subjects who were not taking antibiotics (n = 3,096). (c) ARG_cluster99s were categorized by the combination two factors: whether the cluster's species range contains multi-species or single-species; and whether the ORFs from pathogens and/or non-pathogenic residents were contained. ARG abundance (cpg) values in each sample were then summed by the resulting categories and shown as boxplots. (d) Additionally, the summed cpg values were calculated for each of the four major bacterial phyla found in the stool metagenomes. Affiliation of an ARG_cluster99 into a phylum was determined by the least-common ancestor taxon of the SGBs that contributed ORFs to the cluster. The number of metagenome samples used in c and d: n = 2823 for background resistotype, n = 2272 for FAMP resistotype. In the box plots, the box spans from 25th to 75th percentiles, line gives the median, and the whisker spans from the minimum to the maximum values.

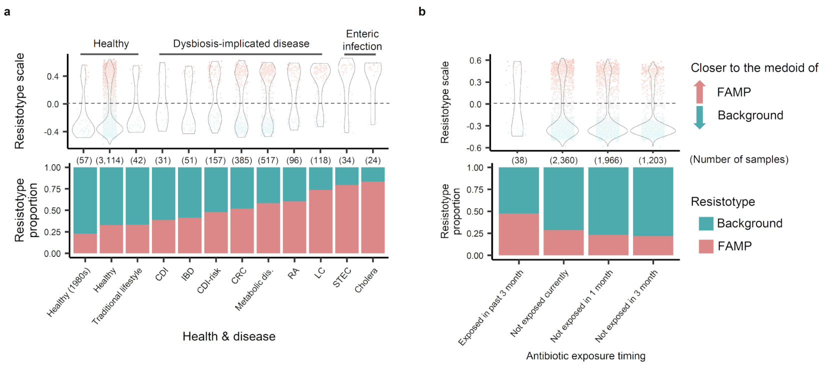

Figure S11: **Frequency of the background and FAMP resistotypes in the subgroups of subjects defined by disease status and antibiotic exposure status.** (a) Resistotype frequencies in the subjects compared across disease status. We used stool metagenome samples from adults that were assigned to resistotype and had disease status metadata (n = 5,260). Disease status of a subject was determined from the original publication that the sample was described in. (b) Resistotype frequencies in the healthy subjects compared across antibiotic exposure status. Starting with the samples in the 'healthy' category described in the panel (a) (n = 3,522), we further restricted to the samples that we could identify either past antibiotic usage within the three months prior to the sampling or confirmable 'antibiotic-free' period of any length prior to the sampling (n = 2,719). Categories for 'not exposed' periods were defined in a redundant way rather than in a non-exclusive way, thus an individual sample belonging to 'not exposed in 3 month' apparently also belong to 'not exposed currently' and 'not exposed in 1 month' categories. Abbreviations used to indicate health and disease states in (A): IBD, inflammatory bowel disease; CDI, Clostridium difficile infection; CDI-risk, the patients diagnosed to be at risk of developing CDI, which is known to be induced by antibiotics; RA, rheumatoid arthritis; Metabolic dis(eases), encompassing type 2 diabetes, hypertension, and fatty liver disease; LC, liver cirrhosis; STEC, shiga toxin-producing *Escherichia coli* infection.

References

[1] R. A. F. Wozniak, M. K. Waldor, Integrative and conjugative elements: mosaic mobile genetic elements enabling dynamic lateral gene flow, Nature Reviews Microbiology 8 (8) (2010) 552–563. doi:10.1038/nrmicro2382.

[2] T. K. Nielsen, P. D. Browne, L. H. Hansen, Antibiotic resistance genes are differentially mobilized according to resistance mechanism, GigaScience 11, giac072 (07 2022). doi:10.1093/gigascience/giac072.

[3] A. N. Zhang, L.-G. Li, L. Ma, M. R. Gillings, J. M. Tiedje, T. Zhang, Conserved phylogenetic distribution and limited antibiotic resistance of class 1 integrons revealed by assessing the bacterial genome and plasmid collection, Microbiome 6 (1) (2018) 130. doi:10.1186/s40168-018-0516-2.
