# Supplementary material for: Population-level impacts of antibiotic usage on the human gut microbiome
Source: Nat Commun. 2023 Mar 2;14:1191. doi: 10.1038/s41467-023-36633-7 (PMC9981903; doi:10.1038/s41467-023-36633-7)
Supplement: Supplementary file 3 — Description of Additional Supplementary Files [file 41467_2023_36633_MOESM3_ESM.pdf]

Description of Additional Supplementary File

sFile Name: Supplementary Data 1

Description: SRA sample accession numbers.

File Name: Supplementary Data 2

Description: Human microbiome samples details.

File Name: Supplementary Data 3

Description: Metagenome-assembled genomes.

File Name: Supplementary Data 4

Description: RefSeq genomes.

File Name: Supplementary Data 5

Description: SGBs identified as human pathogens.

File Name: Supplementary Data 6
Description: ARG family binning and multi-species rates.
