# Supplementary material for: Population-level impacts of antibiotic usage on the human gut microbiome
Source: Nat Commun. 2023 Mar 2;14:1191. doi: 10.1038/s41467-023-36633-7 (PMC9981903; doi:10.1038/s41467-023-36633-7)
Supplement: Supplementary file 5 — Reporting Summary [file 41467_2023_36633_MOESM5_ESM.pdf]

nature portfolio

Corresponding author(s): Christopher Quince

Last updated by author(s): Jan 10, 2023

Reporting Summary

Nature Portfolio wishes to improve the reproducibility of the work that we publish. This form provides structure for consistency and transparency in reporting. For further information on Nature Portfolio policies, see our Editorial Policies and the Editorial Policy Checklist.

Statistics

For all statistical analyses, confirm that the following items are present in the figure legend, table legend, main text, or Methods section.

n/a	Confirmed	
☐	☒	The exact sample size (*n*) for each experimental group/condition, given as a discrete number and unit of measurement
☒	☐	A statement on whether measurements were taken from distinct samples or whether the same sample was measured repeatedly
☐	☒	The statistical test(s) used AND whether they are one- or two-sided<br>*Only common tests should be described solely by name; describe more complex techniques in the Methods section.*
☒	☐	A description of all covariates tested
☐	☒	A description of any assumptions or corrections, such as tests of normality and adjustment for multiple comparisons
☐	☒	A full description of the statistical parameters including central tendency (e.g. means) or other basic estimates (e.g. regression coefficient) AND variation (e.g. standard deviation) or associated estimates of uncertainty (e.g. confidence intervals)
☐	☒	For null hypothesis testing, the test statistic (e.g. *F*, *t*, *r*) with confidence intervals, effect sizes, degrees of freedom and *P* value noted<br>*Give P values as exact values whenever suitable.*
☒	☐	For Bayesian analysis, information on the choice of priors and Markov chain Monte Carlo settings
☒	☐	For hierarchical and complex designs, identification of the appropriate level for tests and full reporting of outcomes
☐	☒	Estimates of effect sizes (e.g. Cohen's *d*, Pearson's *r*), indicating how they were calculated

Our web collection on statistics for biologists contains articles on many of the points above.

Software and code

Policy information about availability of computer code

Data collection	For the retrieval of shotgun metagenome assemblies made available in Pasolli et al 2019 study, no software used other than a mere network retriever Wget. For the retrieval of refseq prokaryotic genome assemblies from NCBI FTP, no software used other than a mere network retriever Wget. For the retrieval of sample metadata for the microbiome assemblies made available in Pasolli et al 2019 study, we used curatedMetagenomicData (3.10). For the retrieval of shotgun metagenomic sequencing raw data we used SRA toolkit (2.10.9) and Kingfisher (https://github.com/wwood/kingfisher-download)
Data analysis	Assembly of the samples outside Pasolli et al 2019 study was performed by Magahit (1.1.1). Protein-coding genes were predicted using Prodigal (2.6.2). ORFs were searched against reference proteins using Diamond (0.9.24). hmmsearch (3.2.1) was used to search CONJscan database. CARD reference database was clustered using CD-hit (4.6), aligned by Muscle (3.8.31), and phylogenetically analyzed by FastTree (2). ARG catalogue was clustered using MMSeqs2 (ad5837b3444728411e6c90f8c6ba9370f665c443). SCG nucleotide sequences were searched against the SGBs using Vsearch (2.4.3). LCA taxonomy was determined by Simple-LCA (github.com/naturalis/Simple-LCA). Plasmid contigs were identified by PlasmidNet (https://github.com/kkpsiren/PlasmidNet). Phylogeny of SGBs were analyzed by Muscle (3.8.31) and IQ-Tree (1.6.11). Scripts for normalized abundance of ARGs and rarefied ARG richness are described in https://github.com/kihyunee/gut_resistotype. Package cluster (2.0.8) was used for partitioning around medoids method. Package outliers (0.14) was used to remove outlier samples. Bipartite network module detection was performed with the package Bipartite (2.16). Negative binomial regression performed by MASS package (7.3). NMDS was performed with vegan (2.6). Statistical tests except otherwise mentioned were performed using R base (4.1.1). Data visualization was made with ggplot2 (3.3.5), ggtree (3.0.4), Cytoscape (3.8.0), with some manual works in Powerpoint (2212).

For manuscripts utilizing custom algorithms or software that are central to the research but not yet described in published literature, software must be made available to editors and reviewers. We strongly encourage code deposition in a community repository (e.g. GitHub). See the Nature Portfolio guidelines for submitting code & software for further information.

Data

Policy information about availability of data

All manuscripts must include a data availability statement. This statement should provide the following information, where applicable:

- Accession codes, unique identifiers, or web links for publicly available datasets
- A description of any restrictions on data availability
- For clinical datasets or third party data, please ensure that the statement adheres to our policy

We did not produce raw sequencing data in this study. Derived data that we produced includes ARG sequence catalogue, sample ARG profiles, and modified CARD database, all can be accessed at https://doi.org/10.5281/zenodo.7383076. The databases used in this study are the NCBI RefSeq (Apr 2019), CARD (Oct 2017), COG (2014), CDDEP ResistanceMap (2019), and WHO report on surveillance of antibiotic consumption 2018.

Human research participants

Policy information about studies involving human research participants and Sex and Gender in Research.

Reporting on sex and gender	This study is a secondary study in which we did not recruit any research subjects herein, but we used the metagenome sequencing data generated in the previous studies that recruited subjects and analyzed their samples. We have gender assignment metadata for the human stool samples, which were collected from the original publications (M = 1,591, F = 2,005, NA = 2,508).
Population characteristics	We don't have genotypic information about the subjects analyzed in this study.<br>The age of the analyzed subjects range from 0 to 91 with median 48 and include 2,658 cases of missing values.
Recruitment	This study is a secondary study in which we did not recruit any research subjects herein, but we used the metagenome sequencing data generated in the previous studies that recruited subjects and analyzed their samples.
Ethics oversight	Organizational or institutional approval was not required for this study as this study is a secondary study in which we did not recruit any research subjects herein.

Note that full information on the approval of the study protocol must also be provided in the manuscript.

Field-specific reporting

Please select the one below that is the best fit for your research. If you are not sure, read the appropriate sections before making your selection.

☐ Life sciences ☐ Behavioural & social sciences ☒ Ecological, evolutionary & environmental sciences

For a reference copy of the document with all sections, see nature.com/documents/nr-reporting-summary-flat.pdf

Ecological, evolutionary & environmental sciences study design

All studies must disclose on these points even when the disclosure is negative.

Study description	We collected publicly avaiable metagenome assembly data of human microbiomes, created a comprehensive catalogue of antibiotic resistance genes (ARGs), and measured relative abundance of these ARGs in each sample. We categorized the catalogued ARGs according to their association with pathogens and commensals, or by their signatures of horizontal gene transfer. Donors of adult stool metagenome samples were categorized according to country of origin, health and disease, and antibiotic usage at the time of sampling. Using these ARGs catalogue and subject information, we explored the population level structures in ARG profiles and evaluated the associations human gut ARGs have with antibiotic consumption rates.
Research sample	Our study is based on public shotgun metagenome sequencing data from human microbiome samples. We did not use specific rationale to determaine the adequate number of samples to address our questions. Instead, we relied on a large set of consistently curated metagenome data, giving the total number of samples 8,972, the highest among the similar studies.
Sampling strategy	Public metagenome data used in this study resulted from DNA sequencing performed in each original study using the total microbial DNA collected from one of the following sample types: human stool, human oral cavity, human vagina, human airway and nasal cavity, or human skin.
Data collection	Sequence data were collected from Pasolli et al 2019 study (10.1016/j.cell.2019.01.001) and NCBI RefSeq. Microbiome sample metadata were collected using curatedMetagenomicData R package and through literature review on the original publications that reported each microbiome sample.
Timing and spatial scale	We focused some of our analyses on adult stool metagenomes and within this scope the geographic origin of the samples were 20 countries after removing the countries represented by less than 10 samples. We focused some of our analyses on adult stool

metagenomes sampled from healthy individuals without current antibiotic usage. Within such scope the geographic origin of the samples were 14 countries after removing the countries represented by less than 10 samples.

Data exclusions	Samples were selected accordingly as described purpose of analysis - e.g., healthy individuals. Additionally, we had formally excluded outliers in case of clustering of overall ARG profiles into distinct resistotypes. In this case, we removed the samples with less than 3 detected ARGs to ensure multivariate analyses. Then we determined the median coordinates from the two major NMDS axes derived from the ARG profiles of all 6,104 adult stools. Euclidean distances from the median coordinates to the individual samples coordinate were subjected to the Grubbs' test. We applied Grubbs' test with p-value cutoff 0.05 to iteratively remove the outlier samples until no outlier is detected.
Reproducibility	As the analyses performed in this study were exclusively computational rather than wet lab experiments, the third persons can reproduce the entire results using the description provided in the methods section and the scripts provided in github.com/kihyunee/gut_resistotype. The factors that could introduce variation in the future reproduction of this study include (1) the improvements in reference ARG database which will change the whole ARG profiles, (2) the increase in the number of RefSeq genomes which will make greater proportion of ARG catalogues assigned into species (from previously unclassified) or multi-species (from previously single-species) and (3) improvements in the coverage across countries and the number of persons sampled.
Randomization	This study does not involve randomization. The samples included in the public metagenome datasets were assigned to healthy control or labeled with certain disease name according to the description provided in the original publication.
Blinding	This study does not involve blinding control. The researchers were aware of the sample origin metadata throughout the study.

Did the study involve field work? ☐ Yes ☒ No

Reporting for specific materials, systems and methods

We require information from authors about some types of materials, experimental systems and methods used in many studies. Here, indicate whether each material, system or method listed is relevant to your study. If you are not sure if a list item applies to your research, read the appropriate section before selecting a response.

Materials & experimental systems

n/a	Involved in the study	
☒	☐	Antibodies
☒	☐	Eukaryotic cell lines
☒	☐	Palaeontology and archaeology
☒	☐	Animals and other organisms
☒	☐	Clinical data
☒	☐	Dual use research of concern

Methods

n/a	Involved in the study	
☒	☐	ChIP-seq
☒	☐	Flow cytometry
☒	☐	MRI-based neuroimaging
